# Repeating Prescriptions—A Remedy Proposed

**Published:** 1870-01

**Authors:** Theodore Griffin

**Affiliations:** Chicago


ARTICLE IV.

### REPEATING PRESCRIPTIONS—A REMEDY PROPOSED.

By THEODORE GRIFFIN, M.D., Chicago.

The prescription repeating abuse has been sufficiently dwelt upon to render it, in all its phases, entirely familiar to every practitioner; so that it is unnecessary to prove the already well-proven fact, that the profession is annually swindled out of many a well-earned dollar, by a universal and persistent resort to this pernicious practice. Our grievances have been well presented, ably and thoroughly discussed, leaving, however, as far as I know, entirely unalluded to, the question of first importance, namely: How shall we remedy this abuse.

It is universally conceded that the patient is not the owner of the written paper prescription, which he conveys to the druggist by direction of the physician, that he may have a remedy for his disease prepared; that, therefore, he has no right to call upon the druggist for its repetition. The prescription is merely the medium of communication between the physician and the druggist who compounds the medicines, and may, when written, be placed in an envelope, sealed up, and addressed to the druggist. It is no more or less than a written communication for facilitating the transaction of business. The patient being merely the bearer of the communication, is not entitled to its use further than especially directed by the physician; which facts preclude the right of the druggist to repeat it without the order of the physician.

The following proposition is presented for the consideration of physicians and druggists, which I believe will, if adopted and faithfully carried out, remedy, as far as can be, under the prescription system of practice, this evil, which deprives us often of a well-merited reward, that we should receive for treatment which is the fruit of years of study and experience, namely: *Let the physician require that the druggist who compounds medicines according to his written directions, return to his office such written directions as he may weekly receive, at the end of each week; numbering the bottles or packages of the patients, and the prescriptions, as he now does, for future reference, if desirable, by both himself and the physician.* The physician should then keep his prescriptions filed, and at all times ready for reference.

The above is the proposition, and my belief is strong that, if it be faithfully carried out on the part of physician and druggist, it will effectually put a stop to this abuse, and spoil the business of numbers of "specialists," who copy from time to time, and practice with the prescriptions of physicians, for certain diseases, which are found on file at our drug stores; elevate the physician in the estimation of his patient, make his advice more deserving of consideration and respect when received, by necessitating his repetition of it, if valuable, and not detract one *iota* from the income of our brethren of the pestle.

In order to secure unanimity of action, let resolutions embodying this idea—or some other one, for a similar purpose—be adopted by our city medical societies, and given to every druggist and physician in the city, with a request that they coöperate with the profession in the enforcement of the same.

The druggists, with whom it is essential that we sustain friendly relations, will, I believe, accept cordially any proposition, which is just to them, that will do away with this vexed question of repeating physicians' prescriptions.

